# A cohort study of factors influencing the physical fitness of preschool children: a decision tree analysis

**DOI:** 10.3389/fpubh.2023.1184756

**Published:** 2023-11-23

**Authors:** Wendi Lv, Jinmei Fu, Guanggao Zhao, Zihao He, Shunli Sun, Ting Huang, Runze Wang, Delong Chen, Ruiming Chen

**Affiliations:** ^1^College of Physical and Health, Jiangxi University of Chinese Medicine, Nanchang, China; ^2^School of Physical Education, Nanchang University, Nanchang, China; ^3^Jiangxi Sports Science and Medicine Center, Nanchang, China; ^4^School of Sport Science, Beijing Sport University, Beijing, China; ^5^School of Physical Education and Sport Science, Fujian Normal University, Fuzhou, China; ^6^PLA Army Academy of Artillery and Air Defense, Nanjing, China

**Keywords:** decision tree, preschool, physical fitness, physical activity, cohort study

## Abstract

**Objective:**

Based on the decision tree model, to explore the key influencing factors of children’s physical fitness, rank the key influencing factors, and explain the complex interaction between the influencing factors.

**Methods:**

A cohort study design was adopted. 1,276 children (ages 3–6) from 23 kindergartens in Nanchang, China, were chosen for the study to measure the children’s physical fitness at baseline and a year later and to compare the physical fitness scores at the two stages. The study was conducted following the Chinese National Physical Fitness Testing Standard (Children Part); To identify the primary influencing factors of changes in physical fitness, a decision tree model was developed, and a questionnaire survey on birth information, feeding patterns, SB, PA, dietary nutrition, sleep, parental factors, and other relevant information was conducted.

**Results:**

The levels of physical fitness indicators among preschool children showed a significant increase after 1 year. The accuracy of the CHAID model is 84.17%. It showed that 7 variables were strongly correlated with the physical changes of children’s fitness, the order of importance of each variable was weekend PA, weekend MVPA, mother’s BMI, mother’s sports frequency, father’s education, mother’s education, and school day PA. Three factors are related to PA. Four factors are related to parental circumstances. In addition to the seven important variables mentioned, variables such as breakfast frequency on school day, puffed food, frequency of outing, school day MVPA, parental feeling of sports, father’s occupation, and weekend breakfast frequency are all statistically significant leaf node variables.

**Conclusion:**

PA, especially weekend PA, is the most critical factor in children’s physical fitness improvement and the weekend MVPA should be increased to more than 30 min/d based on the improvement of weekend PA. In addition, parental factors and school day PA are also important in making decisions about changes in fitness for children. The mother’s efforts to maintain a healthy BMI and engage in regular physical activity are crucial for enhancing the physical fitness of children. Additionally, other parental factors, such as the parents’ educational levels and the father’s occupation, can indirectly impact the level of physical fitness in children.

## 1 Introduction

Early childhood motor development is very active, especially in spatial orientation and coordination. Therefore preschool age is usually named the golden age of motoric. The behavioral habits formed during this period, especially the habit of physical activity (PA) started during this period, have an essential influence on the habit patterns in adulthood (1). Moreover, the development of physical fitness in preschoolers will not only affect the level of fitness in the majority (2, 3) and is even closely related to the risk of disease development in adulthood (4, 5). Therefore, a targeted approach to physical fitness promotion in preschoolers is significant.

However, five times the National Physical Fitness Testing Standard (Children Part) data show that the proportion of children aged 3 to 6 years old who do not reach the qualified level is as high as 6.4 ~ 14.3%. In addition, the physical development of preschoolers shows unevenness in different indicators. Compared with the National Physical Fitness Testing Standard (Children Part) data in 2014, the average levels of height, sitting height, weight, chest circumference, and balance beam walking for both boys and girls in 2020 have increased. Still, the double-leg timed hoping, sit and reach test, and standing long jump are also decreasing. Although the report did not provide a correlation statistical description, the results described are sufficient to attract attention from society and the country (6). To develop a scientific strategy for fostering early childhood fitness and to target the work on early childhood fitness, the prerequisite is to grasp the main influencing factors of early childhood fitness development.

Studies have shown many factors are associated with physical health. PA has a positive effect in enhancing the physical fitness levels of young children (7–10), The researchers discovered a substantial positive association between VPA, MVPA, and physical fitness. These findings imply that promoting MVPA may yield enduring advantages for body composition and physical fitness. Compared to PA, SB has negative effects on health (11, 12), Sedentary behavior is commonly observed among young children, and prolonged sedentary time is associated with higher body fat composition. On the contrary, maintaining a healthy dietary nutrition during early childhood has a positive impact on reducing the risk of obesity and promoting the physical fitness of young children (13, 14). Research substantiated that genetic factors not only play a significant role in determining body type and physical adaptability but also have a crucial impact on the level of physical health and development (15, 16). Some studies have found that sleep plays a crucial role in children’s health, and there is a close association between obesity, sleep, and physical activity in preschool children.

Apart from sleep, several studies have indicated that breastfeeding reduces the risk of childhood overweight and obesity compared to non-breastfed children (17, 18). In the process of child development, the influence of parents on their children cannot be overlooked. There is a correlation between parental-reported physical activity levels of preschool children and objectively measured physical health and body composition. The environment and support provided by parents play a crucial role in promoting the development of children’s motor skills (19, 20). Among the many factors, identifying the key factors influencing changes in physical fitness and grasping their potential interactions have become the focus of academic attention.

Existing studies have mostly used ANOVA, correlation analysis, and logistic regression to explore the degree of influence of different factors on the physical fitness of preschoolers. However, traditional models can be challenging to implement for individuals without a strong statistical background. Furthermore, interpreting the results of these regression models can be complex. as these methods lack deep data mining and decision analysis and are vulnerable to covariance problems (21, 22). In recent years, scholars have used decision trees in data mining techniques to conduct functional explorations in many fields (23–32). As an important classification technique in data mining, decision trees are capable of effectively identifying the main influencing factors of physical changes. They can optimally partition samples based on various types of variables, automatically determining the classification based on the significance of tests, and overcoming collinearity issues. Additionally, decision trees utilize tree diagrams to illustrate the interaction between variables at different levels (21, 28). Decision trees have been applied in beneficial explorations in the fields of physical fitness and exercise training, such as the decision-making of human muscle strength indicators (24), analysis of influencing factors in practical teaching of public physical education courses in universities (26), key influencing factor analysis of overweight and obesity in young children (28), as well as the analysis of factors influencing physical activity in children and adolescents during the COVID-19 pandemic (29).

Given the advantages of decision trees in terms of optimal sample partitioning, overcoming covariance, and mining interaction relationships. We conducted an accurate and visually representative decision tree model, which garnered attention and recognition from scholars. This not only further enhanced the research framework for studying children’s physical fitness but also provided important decision-making references for comprehensive promotion of their physical health (33). However, the decision tree model established in this study was based on cross-sectional surveys, where data was collected by measuring different indicators for individuals at the same time point. Consequently, it cannot establish explicit causal relationships between variables. To further improve the research framework on factors influencing young children’s physical fitness, this study obtained physical fitness data of young children over two consecutive years through a cohort study. The overall physical fitness scores of the same child over the two-year period were compared using age-based scoring. A decision tree was then employed to identify key factors contributing to changes in young children’s physical fitness, offering important evidence-based recommendations for the development of tailored physical fitness promotion strategies suited to the needs of families, kindergartens, and society.

## 2 Materials and methods

### 2.1 Participants

The study was a cohort study, using a whole-group stratified random sampling method. The sampling of participants was based on the public welfare activity of “Concerning the physical fitness of preschoolers and taking care of children’s health” of Nanchang Sports Bureau, China. A total of 23 kindergartens were selected in each of the 6 districts and 3 counties in Nanchang, with a total of 5,870 participants aged 3–6 years old, with an average age of 4.05 ± 0.86 years. All participants were healthy and had no motor impairment. All participants had parental consent to participate in the study and signed an informed consent form. All participants underwent two early childhood physical fitness tests at baseline and 1 year later, during which questionnaires were administered. Daily habits were maintained during the testing period. At the end of the test, those who did not complete the two stages of the physical fitness test and those who did not complete or completed the questionnaire were excluded, and the remaining 1,276 children who participated effectively throughout the test were used as participants for statistical analysis of the data in this cohort study (Table 1).

**Table 1:** The basic information of participants.

Gender	Population/n	Height/cm	Weight/kg
Male	707	110.26 ± 5.98	19.77 ± 3.37
Female	569	108.87 ± 5.68	18.72 ± 2.98

### 2.2 Physical fitness test

The physical fitness test and comprehensive rating were conducted according to the test method and scoring standard of “National Physical Fitness Testing Standard (Children Part)” from China using the designated equipment for national physical fitness testing. The test indexes included physical form indexes and physical quality indexes, including standard weight (reflecting body development and nutritional status), height (reflecting longitudinal skeletal growth), 10-m shuttle run (reflecting body agility), standing long jump (reflecting lower body explosive power and body coordination), tennis throw (reflecting upper body and core muscle strength), double-leg timed hop (reflecting coordination and lower limb muscle strength), sit and reach testing (reflecting trunk and lower limb flexibility), and balance beam walking (reflecting balance ability), with timing accuracy of 0.1 s, measurement accuracy of 0.1 cm, and weight accuracy of 0.1 kg. These 8 indicators were scored separately based on different age groups, with each item having a maximum score of 5 points, totaling 40 points. The comprehensive grading criteria were as follows: excellent (>31 points), good (28–31 points), pass (20–27 points), fail (<20 points). Subsequently, the difference in the overall physical fitness score between the two stages was calculated to determine and analyze the changes in children’s physical fitness, specifically whether there was improvement, no change, or decrease. Taking into account the actual sample conditions and the requirements of the decision tree model used in this study, the target variable (difference in physical fitness scores) was categorized into 2 levels of: “improvement (including no change)” (≥0 points) and “decrease” (<0 points).

### 2.3 Questionnaires

The questionnaire referred to the content of the Questionnaire on Health Behavior of parental and Preschool Children from the Excellent Academic Leaders Program of Shanghai, China (12XD1404500), and used the Delphi method to screen the indicator variables by integrating the opinions of 10 expert teachers from Shanghai Institute of Physical Education, Nanchang University, and Jiangxi Institute of Sports Science. The questionnaire included six primary indicators of young children’s birth information (gender, birth length, birth weight), feeding patterns (feeding mode within 4 months after birth, caregiver, attend early childhood classes), PA (school day SB, weekend SB, school day PA, weekend PA, school day MVPA, weekend MVPA, frequency of outing, frequency of participating in sports organization activities every week), dietary nutrition [fruits, vegetables, candy, carbonated soft drinks, milk, fish and shrimp, eggs, hamburgers or hot dogs, pizza, instant noodles, puffed food (d/w)], breakfast frequency on school day, weekend breakfast frequency [vitamin D, calcium, cod liver oil, zinc (d/w)], sleep (total sleep time, sleep time on school day, weekend Sleep Time, time to fall asleep, frequency of getting enough sleep in a week), and parental factors (age of father and mother, BMI of father and mother, type of registered residence of father and mother, education background of father and mother, income of father and mother, parental occupation, parental working days SB, parents’ weekend days SB, parental frequency of vigorous activity, parental sports frequency in every weekend, parental feeling of sports, marriage and upbringing of biological parents), which were divided into 59 secondary indicators (Table 2). The questionnaire was validity scored by five early childhood health experts, with a mean score of 96.20. The questionnaire was tested for reliability using the retest method, and 812 children tested in three kindergartens were selected to repeat the survey with a 2-week interval, and the intra-group correlation coefficient (ICC) was used to test, yielding a reliability coefficient of 0.86, which met the statistical requirements.

**Table 2:** The basic information of participants (baseline).

Age	Boy		Girl		Total	
	*n*	%	*n*	%	*n*	%
3	162	12.7%	142	11.1%	304	23.8%
3.5	167	13%	132	10.3%	299	23.4%
4	193	15.1%	149	11.7%	342	26.8%
4.5	154	12.1%	125	9.8%	279	21.8%
5	28	2.2%	16	1.3%	44	3.4%
5.5	3	0.2%	5	0.4%	8	0.63%
Total	707	55.4%	569	44.5%	1,276	100%

Questionnaires were distributed to parental of preschool children with the assistance of the Jiangxi Institute of Sports Science, Nanchang Sports Bureau and the kindergartens where the participants were enrolled, and were brought back by parents to be filled out and collected the next day. A professional data entry company processed the collected questionnaires, and the questionnaire results were entered into the database using EpiData software. Double-entry and logical error checking were performed to eliminate those who did not complete the questionnaires or had invalid questionnaire options. The number of valid questionnaires was counted after processing.

### 2.4 Statistical analyses

The results of the physical fitness and questionnaire survey data of preschool children were entered using EpiData 3.1 software with dual input. With the help of SPSS 22.0 data software, the information from physical fitness test and questionnaire survey were matched and integrated to eliminate invalid data. The software IBM SPSS modeler was used to create a decision tree model (parameters to be set as follows: the maximum depth of the tree structure is 5, the minimum number of cases of nodes influencing the change in the physical fitness of toddlers is 100, the minimum number of instances of sub-nodes is 50, and the minimum change value of Gini coefficient is 0.0001), and a 10-level crossover was used to verify the accuracy of the model identification. The Chi-squared Automatic Interaction Detector (CHAID), also known as the chi-square automatic interaction detection algorithm, is a decision tree technique based on adjusted significance testing (Bonferroni test), uses the chi-squared test to evaluate the significance of variable grouping on the target variable and selects the splitting point that maximizes the chi-squared value as the optimal splitting point (21–33). Due to the characteristics of large sample size, multiple indicators, this study used the significance of chi-square tests to automatically determine the grouping variables and split values of the multivariate contingency table, thereby quickly and effectively identifying the main influencing factors. The target variables in the model, which is the change in physical fitness, were divided into 2 categories according to the research needs, with children with improved (or unchanged) physical fitness (≥0) marked as “1” and children with decreased physical fitness marked as “2” (<0). The categorical variables were assigned values, and the best cut-off points for the remaining continuous variable types were identified and split by the decision tree model.

## 3 Results

### 3.1 Results of the physical fitness test

This study collected 1,276 valid samples, of which 707 were boys, accounting for 55.4%; there are 569 girls, accounting for 44.5%. There are 304, 299, 342, 279, 44, and 8 children aged 3, 3.5, 4, 4.5, 5, and 5.5, respectively. The average age is 3.8 ± 0.6 years, respectively, ranging from 3 to 5.5 years. In this study, data comparison revealed that the levels of all eight physical fitness indicators were significantly higher (*p* < 0.01) in preschool children in the second stage compared with the first stage (Table 3), and the scores of sit and reach testing, tennis throw, standing long jump, double-leg timed hop, and balance beam walking were significantly higher (Table 4).

**Table 3:** Influencing factors and assignment table.

Level I indicators	Secondary indicators	Type	Assign value
Birth information	gender	Cla	1 = male, 2 = female
	birth length (cm)	Con	
	birth weight (kg)	Con	
Feeding patterns	feeding mode within 4 months after birth	Cla	1 = breast feeding, 2 = artificial feeding, 3 = mixed feeding
	caregiver	Cla	1 = independent care by parents, 2 = independent care by grandparents, 3 = joint care by parents and grandparents, 4 = joint care by parents and nannies, 5 = joint care by grandparents and nannies, 6 = joint care by parents, grandparents and nannies, 7 = others
	attend early childhood classes	Cla	1 = Yes, 2 = No
	school day SB (h/d)	Cla	1 = ≈0, 2 = 0.5, 3 = 1, 4 = 2, 5 = 3, 6 = 4, 7 = 5, 8 = 6, 9 = ≥7
	weekend SB (h/d)	Cla	
PA and SB	school day PA (h/d)	Cla	1 = ≈0, 2 = 0.5, 3 = 1, 4 = 1.5, 5 = 2, 6 = 2.5, 7 = 3, 8= > 3
	weekend PA (h/d)	Cla	
	school day MVPA (min/d)	Cla	1 = ≈0, 2 = 15, 3 = 30, 4 = 45, 5 = 60, 6= > 60
	weekend MVPA (min/d)	Cla	
	frequency of outing (d/w)	Cla	1 = ≈0, 2 = 1, 3 = 2, 4 = 3, 5 = 4, 6 = 5, 7 = 6, 8 = 7
	frequency of participating in sports organization activities every week	Cla	1 = 0, 2 = 1 ~ 3, 3 = ≥4
Dietary nutrition	fruits, vegetables, candy, carbonated soft drinks, milk, fish and shrimp, eggs, hamburgers or hot dogs, pizza, instant noodles, puffed food (d/w)	Cla	1 = 0, 2 = 1 ~ 2, 3 = 3 ~ 4, 4 = 5 ~ 6, 5 = 7
	breakfast frequency on school day (d/w)	Cla	1 = 0, 2 = 1, 3 = 2, 4 = 3, 5 = 4, 6 = 5
	weekend breakfast frequency (d/w)	Cla	1 = 0, 2 = 1, 3 = 2
	vitamin D, calcium, cod liver oil, zinc (d/w)	Cla	1 = 0, 2 = ≥1
Sleep	total sleep time (h)	Con	
	sleep time on school day	Cla	1 = ≤20:30, 2 = 21:00, 3 = 21:30, 4 = 22:00, 5 = 22:30, 6 = 23:00, 7 = ≥23:00
	weekend Sleep Time	Cla	
	time to fall asleep	Con	
	frequency of getting enough sleep in a week (d/w)	Cla	1 = 4 ~ 7, 2 = 3 ~ 5, 3 = 1 ~ 2, 4 = 0
Parental factors	age of father and mother (y)	Con	
	BMI of father and mother (kg/m^2^)	Con	
	type of registered residence of father and mother	Cla	1 = urban hukou in Nanchang, 2 = agricultural hukou in Nanchang, 3 = urban hukou in other provinces and cities, 4 = agricultural hukou in other provinces and cities, 5 = Hong Kong, Macao, Taiwan and overseas
	education background of father and mother	Cla	1 = junior high school or below, 2 = senior high school or technical secondary school, 3 = junior college, 4 = undergraduate, 5 = master’s degree, 6 = doctor’s degree
	income of father and mother (cny/M)	Cla	1 = ≈0，2 = ≤2000，3 = 2001 ~ 4,000，4 = 4,001 ~ 8,000，5 = 8,001 ~ 15,000，6 = ≥15,000
	parental occupation	Cla	1 = person in charge of state organs, party mass organizations, enterprises and public institutions, 2 = professional technicians, 3 = staff and relevant personnel, 4 = business and service personnel, 5 = production personnel in agriculture, forestry, animal husbandry, fishery and water conservancy, 6 = production and transportation equipment operators and relevant personnel, 7 = military personnel, 8 = other employees
	parental working days SB (h/d) parents’ weekend days SB (h/d)	Cla	1 = ≈0，2 = 0.5，3 = 1，4 = 2，5 = 3，6 = 4，7 = 5，8 = 6，9 = ≥7
	parental frequency of vigorous activity (d/w)	Cla	1 = ≈0，2 = 1，3 = 2，4 = 3，5 = 4，6 = 5，7 = 6，8 = 7
	parental sports frequency in every weekend	Cla	1 = 0，2 = 1 ~ 2，3 = 3 ~ 5，4 = ≥6
	parental feeling of sports	Cla	1 = very like, 2 = quite like, 3 = average, 4 = quite dislike, 5 = very dislike
	marriage and upbringing of biological parents	Cla	1 = normal marriage, 2 = divorced and unmarried, supported by father, 3 = divorced and unmarried, supported by mother, 4 = divorced and remarried, supported by father, 5 = divorced and remarried, supported by mother, 6 = other

Cla: Variable of classification, Con: Variable of continuous, y: year old, M: month, w: week, d: day, h: hour, min: minute, SB: sedentary behavior, PA: physical activity, MVPA: medium and high intensity physical activity, BMI: body mass index, cny: RMB. The “school day” activities only include “outside the school.”

**Table 4:** Physical fitness of preschoolers.

Stage	Height (cm)	Weight (kg)	Sit and reach (cm)	Tennis throw (m)	Standing long jump (cm)	10-m shuttle run (s)	Double-leg timed hop (s)	Balance beam walking (s)
Baseline	103.5 ± 5.8	17.7 ± 3.0	10.7 ± 4.1	3.0 ± 1.2	63.9 ± 21.8	9.3 ± 2.0	9.2 ± 4.5	14.2 ± 9.6
12 months	109.6 ± 5.9^*^	19.3 ± 3.2^*^	11.7 ± 4.6^*^	4.2 ± 1.5^*^	82.2 ± 19.0^*^	8.2 ± 1.3^*^	6.7 ± 2.5^*^	11.9 ± 8.1^*^

* *p* < 0.01.

### 3.2 Construction of decision tree model

**Figure 1:**
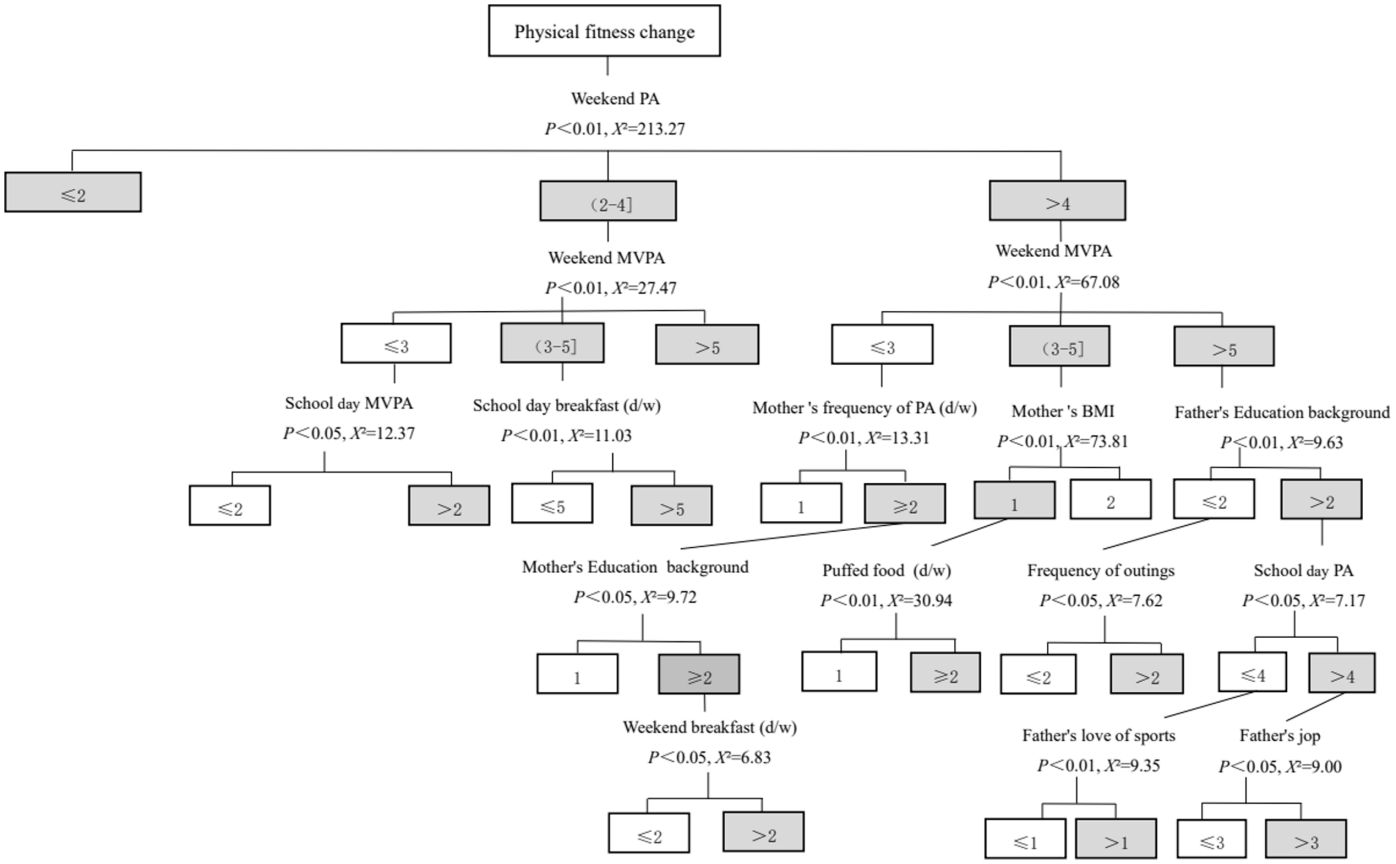
The model of CHAID. Layer 1 – weekend PA: 2 = 30 min, 3 = 1 h, 4 = 1.5 h; Layer 2 – weekend MVPA: 3 = 30 min, 4 = 45 min, 5 = 1 h; Layer 3 - mother’s BMI: 1 = <24.17, 2 = ≥24.17; Layer 3 - frequency of mother’s sports: 1 = 0 times; 2 = 1 time; Layer 3 – school PA: 2 = 30 min; Level 3 - Father’s education background: 2 = vocational high school/technical secondary school/technical high school or technical secondary school; Level 4 – Education background of mother: 1 = junior high school or below.

The decision tree model created in this study has 5 layers and 41 leaf nodes (Figure 1), and the decision tree model shows that the root node variable is weekend PA. Therefore weekend PA is the most important influencing factor on the change in physical fitness of preschool children. The 1st layer of the tree structure splits the nodes according to weekend PA. This layer has three nodes “>1.5 h/d,” “30 min ~ 1.5 h/d,” and “≤30 min/d,” where the node weekend PA “>1.5 h/d” (85.1%) had the highest rate of physical fitness improvement for preschoolers, followed by “30 min ~ 1.5 h/d” (77.0%), while “≤30 min/d” (30.4%) was the lowest. Weekend PA “>1.5 h/d” (85.1%) was very significantly different from that of children with weekend PA “30 min ~ 1.5 h/d” (77.0%) and “≤30 min/d” (30.4%; *p* < 0.001). It is clear that preschoolers with longer weekend PA have better physical fitness improvement.

The three split nodes in the layer 2 leaf nodes were all weekend MVPA. Combined the analysis with the layer 1 nodes, it was found that in the group with weekend PA “>1.5 h/d,” Weekend MVPA node “>5” (>1 h/d; 86.3%) and node “(3–5)” (30 min ~ ≤1 h/d; 94.1%) had a significantly higher rate of physical fitness improvement than node “≤3” (≤30 min/d) toddlers (68.6%; *p* < 0.001).

Branching downward from the second layer of leaf nodes, the branching characteristics of different nodes are quite different. The third level leaf node variables include mother’s sports frequency, mother’s BMI, father’s education, and school day MVPA. The branch weekend MVPA nodes “>4” (>1.5 h/d) were classified based on the frequency of mother’s PA, and the weekly frequency of mother’s PA nodes “≥2” (1 and more times per week; 71.6%) had a significantly higher rate of fitness improvement than those with node “1” (0 times per week; 23.1%; *p* = 0.001). The downward branch of “≥2” (1 and more times per week) on frequency of mother’s PA nodes is the mother’s educational background, and the last branch is weekend breakfast. The branch weekend MVPA node “(3–5)” (30 min ~ ≤1 h/d) with weekend PA node “>4” (>1.5 h/d) was split into “1” and “2” according to mother’ s BMI, children with mother’ s BMI node “2” (<24.17; 98.0%) had a significantly higher rate of fitness improvement than those with maternal BMI node “1” (≥24.17; 63.2%; *p* < 0.001). Mother BMI node “1” (≥24.17) branches down to buffered food.

The branch with weekend PA node “>4” (>1.5 h/d) was the weekend MVPA node “>5” (>1 h/d), which was classified according to the father’s education, and the father’s education “≤2” (vocational high school/junior high school/technical high school or junior high school and below) were significantly higher (95.1%) than those with “>2” (bachelor’s/master’s/doctoral degree; 82.2%). Among children whose fathers’ education was node “>2,” preschoolers with school day PA “>4” (>1.5 h/d; 91.0%) had a significantly higher rate of physical fitness improvement than preschoolers with school day PA “≤4” (≤1.5 h/d; 76.4%). The downward branches of school day PA “≤4” (≤1.5 h/d) and school day PA “>4” (>1.5 h/d) are father’s love of sport and father’s occupation, respectively.

Combining the nodes in stratum 1, it was found that among preschoolers with weekend PA “30 min ~ ≤1.5 h/d,” stratum 2 weekend MVPA node “>5” (>1 h/d; 76.2%) and node “(3–5)” (30 min ~ ≤1 h/d; 94.7%) were significantly higher (*p* < 0.001) than preschoolers with node “≤3” (≤30 min/d; 65.8%). In addition to weekend PA, weekend MVPA, mother’s BMI, frequency of mother’s sports, father’s education, mother’s education, and school day PA as the main influencing factors, school day breakfast, puffed food, days out with children, school day MVPA, father’s love of sports, father’s occupation, and weekend breakfast were all statistically significant leaf node variables.

### 3.3 Ranking of key influencing factors

The CHAID model generated by IBM SPSS modeler software was used to rank the importance of each variable based on the decision tree node positions as well as the values. A total of seven variables that were important for the change in physical fitness of preschool children were screened. Each variable was ranked in order of importance: weekend PA, weekend MVPA, mother’s BMI, mother’s sports frequency, father’s education, mother’s education, and school day PA (Table 5).

**Table 5:** Physical fitness score of preschoolers.

Stage	Height weight score	Sit and reach (cm)	Tennis throw (m)	Standing long jump (cm)	10-m shuttle run (s)	Double-leg timed hop(s)	Balance beam walking (s)
Baseline	4.0 ± 1.5	3.1 ± 1.2	2.2 ± 0.9	2.9 ± 1.2	2.7 ± 1.0	1.6 ± 0.9	2.7 ± 1.1
12 months	4.1 ± 1.5	3.4 ± 1.2^*^	2.3 ± 1.0^#^	3.3 ± 1.0^*^	2.8 ± 1.0	3.6 ± 1.2^*^	2.4 ± 1.0^*^

* *p* < 0.01.

# *p* < 0.05.

### 3.4 Evaluation of decision tree model

A 10-layer cross-validation model was used to identify the accuracy, and the model’s accuracy was 84.17%, indicating that the model was effective (Table 6).

**Table 6:** Recognition accuracy of the model.

Name	*n*	%
Model accuracy	1,074	84.17
Model error	202	15.83
Total	1,276	100

## 4 Discussion

In this study, by tracking the measurement of preschool children’s fitness levels and changes. It was found that overall, various aspects of preschool children’s physical fitness improve with age. Through in-depth analysis using decision trees, the seven most significant influencing factors were identified. These factors exert their influence through both direct and indirect pathways. Among them, PA and parental factors were identified as key factors influencing the changes in preschool children’s physical fitness. This finding is consistent with the results of most relevant studies, and once again suggests that the importance of promoting PA in children’s 24-h behavior should be emphasized, and the role of families in promoting PA should be played. This result also proves the effectiveness of the decision tree model in identifying the influencing factors of preschool children’s physical fitness.

### 4.1 Construction of decision tree model

Many previous studies have analyzed the factors affecting children’s physical fitness from different perspectives, mainly from personal factors (gender, age, physical activity, race, etc.) (34, 35), family factors (parents’ physical activity, children’s nutrition, etc.) (17, 18, 36), community factors (built environment, community support, etc.) (37, 38) and other aspects. These studies mainly analyze the effects of single or multiple factors, and it is easy to overlook the impact of other factors on the physical fitness of young children. Indeed, there is a relationship between the effects of various factors that overlap or cancel out, that is, the interaction between various factors has an interactive effect on the physical fitness of preschool children. In fact, the improvement or decrease of preschool children’s physical fitness and the influencing factors do not show a simple linear relationship. After segmenting the samples for different types of variables, the close relationship between independent variables and dependent variables in a specific type of sample can be explored. For example, in a study by Zhenya et al. (39), the dose effect of 24-h isochronous replacement of motor behavior time and changes in body mass in preschool children was investigated using component data analysis. On the contrary, as the time of isochronous substitution of LPA, SB and sleep for MVPA increased, the level of body mass decreased rapidly and the decrease was greater than the increase in the other three behaviors of MVPA isochronous, and these findings corroborate the existence of complex interactions among the independent variables. Decision tree, as an important classification method in data mining, is characterized by optimal segmentation of samples based on different types of variables, and automatic classification of samples based on the significance of the test.

The CHAID model in decision tree is used in this study, and the constructed CHAID model is 5 levels high, divided into 41 leaf nodes, and has a branching tree. The accuracy of the model reaches 84.17%, which meets the needs of this study.

### 4.2 Decision tree analysis of factors influencing physical fitness

Weekend PA, weekend MVPA, mother’s BMI, mother’s frequency of PA, father’s education, mother’s education and school day PA were the seven most important influencing factors. Besides, school day breakfast, puffed food, taking the child out for a few days, school day MVPA, father’s love for sports, father’s occupation, and weekend breakfast all influenced different nodes of body mass change in preschool children. From the above-influencing factors, it was found that PA and parental factors are the key to influence the evolution of body mass in preschool children.

#### 4.2.1 Physical activity

Many studies have confirmed that PA (including exercise) is an important factor influencing changes in fitness levels. A systematic review study exploring school-based PA interventions found (40) that the included high-quality RCTs confirmed that PA interventions are effective in improving physical fitness and reducing skin-fold thickness in children and adolescents aged 5–18 years. Not coincidentally, another meta-analysis found that school-based PA interventions were associated with a significant small increase in cardiorespiratory fitness (CRF) in children aged 3–12 years (Hedges’ g = 0.22; 95% CI 0.14 to 0.30; *p* < 0.001). According to subgroup analysis, the increase in CRF was significant in girls (Hedges’ g = 0.25; 95% CI 0.13 to 0.37; *p* > 0.001) (41). The findings of these studies undoubtedly provide an important reference for young children’s physical fitness promotion and further confirm the important role of PA in enhancing young children’s physical fitness, consistent with the results of the present study. Investigating the mechanism, cardiorespiratory fitness, regular PA induces molecular adaptations in the cardiovascular system. The increased demand for oxygen during exercise activates signaling pathways that promote angiogenesis, or the growth of new blood vessels. This process involves the release of factors such as vascular endothelial growth factor and fibroblast growth factor, which stimulate the formation of new capillaries, enhancing blood flow to the muscles. Additionally, exercise activates pathways that signal the heart to undergo hypertrophy, leading to increased heart muscle mass and improved cardiac function. In addition, other indicators of physical fitness, such as height, weight, muscle strength, and muscle endurance, have gradually been discovered through relevant mechanisms. However, it is worth noting that most of these mechanism studies are based on adults or children and adolescents, which can provide ideas for the possible mechanisms of PA improving physical fitness in young children. However, further exploration is still needed for the relevant mechanism research in preschooler.

This study found that weekend PA and weekend MVPA were two key factors affecting preschoolers’ physical fitness, highlighting the importance of performing activities on weekends. This is because most preschoolers follow their teachers on school days as required in the school, and therefore their PA levels are all within a certain range. On the other hand, preschoolers have relatively more choices during the weekend, which may lead to more screen time and possibly more time spent outdoors. The behavior of young children in kindergarten is basically carried out under the supervision of teachers, and whether it is SB or PA, it is mainly structured. However, with the trend of using electronic products at a younger age, more and more young children are addicted to electronic products. Once parents fail to supervise during weekends, young children may use electronic products for a long time. On the contrary, parents consciously taking their children out for activities and limiting screen time will be beneficial for the development of children’s physical fitness. The difference in PA between school days and weekends was also found in Meimei ji ‘s research (42) on Chinese young children. Similarly, the findings of this study are consistent with that of scholar Zhao, who found that weekend PA is the key influencing factor for children’s physical development ranking first (33). The study also mentioned that it may be because during the semester of this study, compared to the more regular lifestyle on school days, weekend children had more freedom in choosing physical activities and sedentary behavior, which had a greater impact on their physical fitness. This shows that adequate attention should be paid to preschoolers’ weekend PA, which includes weekend MVPA. In addition, the 6th influencing factor is school day PA, which appears in both the 2nd and 3rd levels of the branch. Therefore, the PA factors of fitness changes in preschool children include not only weekend PA, but also school day PA is an important factor. Both of the previous studies were conducted at kindergarten sites. As the place where children spend most of their day, the kindergarten curriculum on school days is critical, and adequate PA is critical for preschoolers’ physical fitness. This evidence further confirms and adds to the significance of PA in the physical fitness of children and adolescents, including preschoolers.

In addition, the performance characteristics of PA in preschool children have been a hot topic of interest for researchers. The decision tree model showed that preschoolers with weekend PA “>1.5 h/d” had significantly higher fitness levels than those with “30 min ~ 1.5 h/d” and “≤30 min/d. In addition, preschoolers with MVPA “>1 h/d” on the downward branching weekend also improved significantly more than preschoolers with MVPA “≤30 min/d.” The findings of this study are generally consistent with the recommendations of national PA guidelines for young children, with the Australian (43), Canadian (44) and WHO PA guidelines for children referring to no less than “1 h/d” of active play (45), and similarly the Chinese Guidelines for Exercise in Preschool Children (3–6 years) recommending a specific duration of no less than “1 h/d” of MVPA cumulatively (46). These guidelines were developed through a comprehensive search of literature related to preschool children in various fields, combined with the physiological and psychological characteristics, and repeatedly discussed by scholars in various fields, which have essential reference and practical significance. The splitting point value at the root node of the decision tree model is consistent with the present study, that is, MVPA is a vital factor influencing the fitness changes of preschool children, and at least “1 h/d” is a critical splitting point. The difference is that this study found that weekend PA “>1.5 h/d” is a critical point for improving the physical fitness of preschool children, which is lower than the recommended amount of PA in each guideline. The possible reason is that the physical activity level of Chinese children is generally low (42), and children who exceed this threshold can have a significant improvement in their physical fitness.

#### 4.2.2 Parental factors

From the tree diagram of the decision tree, it is clear that both level 1 and level 2 are related to PA, and from level 3 parental factors appear. Parental factors, as indirect influences, may impact changes in preschool children’s physical fitness through direct factors. This study found that mother’s BMI, fequency of mother’s PA, father’s education and mother’s education exerted significant influence in the change of body mass of preschool children. Preschoolers whose mothers’ BMI was greater than 24.17 had a significantly higher decrease in body mass than preschoolers whose mothers’ BMI was less than 24.17. Studies have shown that the effect of mothers’ BMI on preschool children is mainly in the areas of preschool children’s growth and development, diet and exercise habits. On the other hand, overweight and obese mothers have influence on preschoolers’ lifestyle in terms of diet and PA, especially mothers as the main caregivers of preschoolers, and their lifestyle behavioral habits are obvious on the habit formation of preschoolers. A study by Li et al. (47), which explored the effect of parental overweight/obesity on overweight/obesity in children and adolescents, found that children and adolescents whose mothers were overweight or obese were 2.74 times more likely to be overweight and obese than children whose parents were neither overweight/ obese. As the primary caregiver of preschool children’s eating behavior, the mother’s BMI status is related to her eating habits and health perceptions. These differences in habits and perceptions can lead mothers to adopt different feeding practices, affecting the eating behavior habits of their preschool children and, consequently, their body shape. In addition to the influence of acquired factors, some studies have suggested that parental genes are an important factor affecting the physical fitness of young children. These studies have found that innate genes have a certain impact on the physical fitness of young children (48), but some studies have also suggested that the degree of genetic influence is limited (49). However, in the process of developing children’s physical fitness, the acquired influence can be changed and has a profound impact (50). Therefore, it is necessary to pay attention to the impact of family environment on the physical fitness of young children.

The results of this study also reaffirm the positive effect of mothers’ frequency of physical activity participation on the development of PA habits and physical fitness of preschool children. The present study also found that preschool mothers’ participation in sports once a week or more was significantly higher than preschool children’s physical fitness who participated in sports 0 times a week. The influence of mothers’ sports habits on their children is undoubtedly subtle. Mothers who participate in sports regularly can create a good PA atmosphere for their children and act as role models in developing their children’s sports habits. These sports habits play an important role in the promotion of physical fitness in preschool children. Zhao et al. (51) explored the differences in physical fitness and PA between overweight and normal weight preschoolers and the relationship between their parental and children’s PA levels, and found that maternal PA was positively correlated with their children’s PA levels, and overweight parents were positively correlated with their children’s overweight. Scholars Rhodes et al. (52) explored the effect of a family-based intervention on improving preschool children’s MVPA and fitness levels through a randomized controlled trial. The study population was divided into a Family PA Planning Plus Information/Education and an Education Information Only group, and it was found that preschoolers in the intervention group had significantly improved MVPA at both the 6 and 13-week measures compared to the control group, and also had significantly improved cardiorespiratory fitness. Thus, family-based facilitation strategies, such as the involvement of mothers in their children’s activities, may be an effective measure to improve preschoolers’ fitness levels.

In addition, parental education and father’s occupation influenced changes in physical fitness in preschool children. The results of the present study are similar to those of a foreign study on adolescents. Finger et al. (53) investigated 5,251 German adolescents aged 11–17 years and found that higher levels of parental education were associated with better aerobic fitness (PA). Higher levels of parental education were found to be associated with higher levels of PA in girls. The reason for this is that parents whose work status is predominantly SB, especially those with higher education, may exercise more in their leisure time and may have stronger expectations to motivate their children to exercise with them (54); in contrast, parents whose work is physically demanding (a group that is mostly less educated) may be less active in their leisure time, as they usually stay at night or on weekends at home, using media for entertainment and relaxation. Parental education does not directly affect the level of fitness of preschoolers, but may in turn affect the change in fitness of preschoolers by increasing PA. In a study that explored the relationship between parental education and PA from another perspective, Xiu et al. (55) found that the total number of parents with educational attainment of junior high school and below had only 6.2% of their children participating in various special classes, while the total number of parents with educational attainment higher than junior high school was as high as 93.8%. In addition, Wang et al. (56) explored the relationship between fathers’ occupation and children’s PA and SB. They found that students whose fathers were agricultural workers reported lower rates of physical activity.

In contrast, fathers with occupations such as clerks, police officers, and military personnel reported higher rates of PA in their children. In addition, fathers whose occupations were casual workers/layoffs and urban farmers reported higher rates of “watching TV/video,” while fathers whose occupations were police and military, and professionals and technicians reported lower rates. The present study also found that preschool children whose fathers’ occupations were state agencies, professionals, clerks, and related personnel had significantly higher physical fitness than preschool children whose fathers were employed in agriculture, animal husbandry, or fishing. The reason for this may be related to family lifestyle and environmental impacts. Engaging in professions such as agriculture, animal husbandry, and fishing may mean more physical labor and fewer opportunities to participate in aerobic or physical exercise. The occupation and nature of work of parents may affect their time arrangement and lifestyle, affecting the opportunities and quality of their children’s participation in sports activities. In contrast, fathers who are engaged in state organs, professional technicians and clerks may have more time and opportunities to encourage their children to engage in sports activities and provide a better sports environment. In summary, based on current research analysis, parents’ educational background and occupation may affect the physical fitness level of young children by influencing their PA.

## 5 Strength and limitations

Through a one-year cohort study, it illustrates the causal relationship between the characteristics of physical change and development and various factors. The decision tree model is used to find the main influencing factors, and the complex interaction between influencing factors is discussed. This provides more practical reference for children’s physical fitness promotion work, and also provides research basis for future research. The limitation of this study is that the measurement of PA and SB time in young children is a subjective questionnaire, which may have some bias compared to the objective measure.

## 6 Conclusion

Through the decision tree model, a total of 7 critical influencing factors for changes in preschool children’s physical fitness were screened. The top 2 factors in order of their importance were weekend PA and weekend MVPA, followed by mother’s BMI, mother’ s sports frequency, paternal education, mother’ education, and school day PA, of which 3 factors were related to preschool children’s PA and 4 factors were related to preschool children’s parental factors. The parental sports habits, body shape, and Feeding patterns have important impacts on the changes in preschool children’s physical fitness.

The results of the tree diagram suggest that to improve the physical fitness level of preschool children, the weekend MVPA should be increased to more than 30 min/d on improving the weekend PA. Mothers with appropriate BMI and higher sports frequency also play an important role in improving the physical fitness of preschool children. Other parental factors such as parental education and father’s occupation are also influencing factors for changes in children’s physical fitness. In addition, parental factors and school day PA have decisional implications for changes in physical fitness in preschool children of different level types.

Through the decision tree model, a total of 7 critical influencing factors for changes in preschool children’s physical fitness were screened. The top 2 factors in order of their importance were weekend PA and weekend MVPA, followed by mother’s BMI, mother’s sports frequency, father’s education, mother’ education, and school day PA, of which 3 factors were related to preschool children’s PA and 4 factors were related to preschool children’s parental factors.

The duration of the present cohort study was 1 year. Although the decision tree model could find the main influencing factors, a follow-up study with a longer period might explain more accurately the influence of different factors on the change of physical fitness of preschool children, so the follow-up study period of the subsequent design could be extended. In addition, the decision tree model demonstrates the complex interaction between different influencing factors through tree diagrams, future studies can conduct comprehensive intervention experiments based on the results of this study to integrate many factors affecting preschool children’s physical fitness for empirical interventions to clarify further and identify key influencing factors, so that more research results will be extended to kindergartens and educational institutions to improve and refine preschool, this will improve the evaluation and service system of children’s physical health. At the same time, the decision tree model is suitable for large-scale, large-group, and large-sample research, and has some prospects for application not only in the field of preschool children’s physical fitness research, but also in the whole field of physical fitness testing, so that more decision tree results can provide scientific and reasonable health advice for different age groups, and target health problems to ultimately achieve the purpose of improving physical health and human quality of life.

## Data availability statement

The original contributions presented in the study are included in the article/supplementary material, further inquiries can be directed to the corresponding author.

## Ethics statement

The studies involving humans were approved by Jiangxi University of Chinese Medicine Ethics Commit/Jiangxi University of Chinese Medicine. The studies were conducted in accordance with the local legislation and institutional requirements. Written informed consent for participation in this study was provided by the participants’ legal guardians/next of kin.

## Author contributions

WL and GZ were responsible for the design of the study. JF and SS provided policy support for the study. ZH, TH, RW, DC, and RC collected the data. WL and ZH has conducted all the analysis and written the first draft of the paper. All authors contributed to the article and approved the submitted version.
